# Warming Churches

**Published:** 1859-02

**Authors:** 


### WARMING CHURCHES.

Many an excellent clergyman has lost his voice, and eventually his life, by preaching in a cold, damp, and close church; and multitudes of people have been made invalids for months and years, and have prematurely died, from sitting in churches insufficiently warmed in winter time.

The atmosphere of any building closed for six days in the week becomes unfit for respiration in summer as well as winter by reason of its damp, heavy closeness. It requires several days for the cold and damp to get into a closed house, and a much longer time for it to get out. Hence, after several days of very severe weather, it may be sultry—even uncomfortably warm in riding, walking, or any other slight effort, and no fire is deemed necessary; on the contrary, the air of the church seems, on first entering, to be refreshingly cool, but has, nevertheless, sowed the seeds of untimely death in multitudes; for, remaining still for a couple of hours, the body becomes chilled through and through, to be followed by fever, pleurisy, inflammation of the lungs, or other dangerous forms of disease.

Many country churches are heated by stoves, which, on cold days, are kept red hot, roasting those who are near, leaving the more distant ones to freeze.

These difficulties may be easily avoided by a little knowledge and attention, which may be illustrated by stating the practice of the sextons of our city churches—or, to be more specific, the practice of the sexton of the church which we attend in Fifth Avenue, Mr. Culyer, who will doubtless be surprised to find his name in print; but as the health and lives of a thousand people are in his custody every winter's day, and as we have not in the course of years ever noticed the building too hot or too cold, his fidelity to duty, and his intelligence in this regard, merits a public notice. A thermometer is kept about five feet above the floor, about half-way between the door and the pulpit. The heat is made to reach fifty-five degrees of Fahrenheit at the time the service is about commencing. With the same heat in the furnace, it is raised to sixty by the warmth imparted from the bodies of the congregation. The fires are not built, as in country churches, on Sabbath morning, but early on Saturday morning, and are kept pushed for twenty-four hours, with a proper opening of doors and windows to secure a thorough airing of the whole building. If the weather is intensely cold, the fires are built early on the Friday morning preceding the Sabbath.

In summer time, the doors and windows are opened at daylight to let in the cool air, and at ten are closed to keep it in. Thus, by these simple arrangements, the building is delightfully cool in midsummer; while, on a zero day, we have the soft and balmy warmth of a southern clime.

